# Developing new therapeutic approaches for rheumatoid arthritis: the continuing challenges of clinical assessments

**DOI:** 10.12688/f1000research.8812.1

**Published:** 2016-08-17

**Authors:** David L. Scott

**Affiliations:** 1 King's College London, London, SE5 9RJ, UK

**Keywords:** rheumatoid arthritis, clinical assessment, intensive treatment strategies

## Abstract

The management of rheumatoid arthritis has changed dramatically over the last three decades. Improvements in clinical assessment have been a key driver of these changes. However, in the last five years, three areas of unresolved uncertainty have dominated specialist thinking in the field. These challenges comprise identifying the optimal management target, determining how best to reach this target by using intensive treatments, and individualising management because not all patients need or respond to identical treatments. The key problem that links each of these areas is balancing different types of evidence and is most readily appreciated in relation to treatment intensity. Giving more intensive therapy improves outcomes but also increases risks and, with biologic treatments, substantially increases drug costs. Specialists and healthcare funders need to agree on how best to rationalise optimal care for patients with what is most effective and safe and what is affordable.

## Introduction

Historical treatments for rheumatoid arthritis (RA) used thirty to forty years ago were limited and outcomes were poor. Persisting joint inflammation and increasing disability caused major health problems. These existing treatments also often led to significant toxicities. As a consequence, patients’ quality of life was reduced and their lives were often shortened.

Two inter-related developments changed this historic scenario. The end results were greatly improved clinical outcomes. The first improvement was identifying new drugs and new treatment strategies. The second change was developing new and better ways to assess RA and define the benefits of treatment. Whilst therapeutic innovations are widely recognised, the importance of assessments has received less emphasis. However, without better assessment methods, the ability to identify new drugs would have been greatly reduced.

### Historical changes in assessments

Before 1990, there were multiple ways to assess RA. Approaches included recording the duration of morning stiffness, measuring finger sizes using jewellers’ rings, and recording grip strength using modified sphygmomanometers. There was no sensitive or agreed way to assess the impact of RA on erosive joint damage, physical function, or quality of life. Finally, recording adverse events was difficult, and the toxicities of many early drugs including steroids were not fully understood when these treatments were introduced.

The situation changed substantially with the development of an internationally agreed core data set to assess disease activity ^1^. This change was linked to the introduction of summary measures of response such as the disease activity score for 28 joints (DAS28) ^2^ and the American College of Rheumatology (ACR) response criteria ^3^. There were also substantial advances in assessing disability using summary measures such as the health assessment questionnaire (HAQ). Assessing structural changes in joints by making better use of conventional X-rays and adopting new imaging modalities such as ultrasound and magnetic resonance imaging has also been important. Finally, understanding the complexities of drug toxicities led to the establishment of large national registries. They provided long-term real-world information about the potential toxicities of existing and new drugs.

### Historical changes in drug treatments

Until the 1980s, the treatment of RA involved treating symptoms with analgesics and non-steroidal anti-inflammatory drugs, giving non-responders disease-modifying drugs that were either toxic (such as gold) or relatively ineffective (such as hydroxychloroquine), and using steroids as needed to control inflammation, knowing that their toxicity would risk long-term problems.

Since then, three main developments have occurred. Firstly, more effective and less toxic disease-modifying drugs including methotrexate, sulfasalazine, and leflunomide became available ^4^. Secondly, starting with tumour necrosis factor (TNF) inhibitors, a range of both highly effective and relatively safe biologic agents was developed ^5^. Finally, in recent years, new orally effective kinase inhibitors, particularly JAK inhibitors, have been developed ^6^.

Improved assessment methods facilitated these developments. Summary responses such as DAS28 and ACR responders reduced the sample sizes needed in trials and provided comparative data across different treatments. Assessing the progression of erosive damage showed treatments were genuinely disease modifying. Measuring disability and quality of life enabled the development of cost-effectiveness methods, which is important to help justify the use of high-cost biologics. Finally, understanding the risks of drug toxicity was vital in enabling new treatments to be used without undue concern.

### Recent challenges

This historical background has led to three current challenges in the management of inflammatory arthritis. All of them reflect the way in which clinical assessments and drug treatments developed over the last three decades. Each of them remains unresolved. These challenges relate to identifying the optimal treatment target, determining how best to reach this target by using intensive treatments, and the need to individualise management because not all patients need or respond to identical treatments.

## Present uncertainties

There are three main uncertainties about the future best management of RA. These comprise the optimal management target, the most suitable intensive treatment strategies, and the role of bespoke care.

### Management targets

Patients want their illnesses to be cured. In long-term diseases like RA, in which a cure seems unachievable, they would like the equally acceptable alternative of disease remission. When patients with arthritis achieve remission, they have less disability and a better quality of life. As a consequence, remission seems a highly appropriate treatment target. A whole treatment movement has grown out of this approach – “Treat To Target” – in which treatment is increased until patients achieve the defined target ^7^. The growing focus on early treatment of inflammatory arthritis has been associated with an increased emphasis by patients on being able to return to normality as soon as possible ^8^.

One central research issue, which remains open to debate, is what the target should be. Remission appears to be the most rational target. However, there are multiple ways of defining remission, and it is uncertain which definition is optimal in clinical practice. An associated theme is whether it is preferable to aim for patients achieving deep and sustained remissions or whether it is better to have modest levels of remission for short periods of time, or even to have sustained low-disease-activity levels.

There are trade-offs between achieving optimal remissions and maximising the numbers of patients who are able to reach the therapeutic target. The deeper and more sustained the remission, the fewer patients likely to be able to reach it. In contrast, achieving low-disease-activity levels is less beneficial for individual patients but substantially more patients can achieve this target.

The remission criterion most widely used as a treatment target is DAS28 remission ^9^. With modern treatments, particularly in patients with early RA, up to 50% of patients can achieve DAS28 remission. A substantial theoretical limitation of DAS28 remissions is the major impact of erythrocyte sedimentation rate (ESR) levels on achieving it. At low levels of DAS28, the ESR contributes 70% or more of the score. This means that the chance of obtaining a DAS28 remission is particularly dependent on controlling the ESR.

Alternative remission criteria, such as remission using the simple disease activity index (SDAI), appear more rational ^10^. This measure is easier to calculate and is not subject to the impact of the ESR in determining remission status. SDAI combines joint counts, global assessments, and C-reactive protein levels rather than the ESR. SDAI remissions are supported by international collaborative groups, which consider it preferable to DAS28 remissions. Currently, SDAI is less often used in routine practice than DAS28. Whether this will change over time is uncertain.

An interesting variant on the approach to assess the severity of synovitis is the clinical disease activity index (CDAI), which combines joint counts with global assessments and has no laboratory assessment of inflammation ^10^. CDAI remissions may be particularly useful in assessing the efficacy of treatments that have less impact on laboratory measures of inflammation. There are other definitions of remission, including the more robust but difficult-to-achieve Boolean remission ^11^, which is rarely adopted in clinical practice.

Sustained remission and drug-free remission are two additional facets of remission. There are no widely agreed criteria for how long sustained remission should last, but some experts have suggested that 6 months appears to be reasonable. The longer remission lasts, and particularly if patients have been able to stop anti-rheumatic drug treatment, the better the outcome for patients.

One limitation of targeting remission is that it places controlling the features of joint inflammation over and above other assessments, such as disability and joint damage. The other limitation is that it avoids targeting other outcomes that may be more important for patients. For example, targeting other symptoms such as pain and fatigue, which are of consequence to patients, may be equally beneficial. Finally, remission may be the best target in early disease, but in established RA, when there may already be some irreversible damage to joints, it may be less relevant and its benefits less obvious.

### Intensive treatment strategies

There are two ways to deliver intensive treatment in RA. Firstly, two or more conventional disease-modifying drugs can be used concurrently, with or without glucocorticoids. Secondly, biologics can be combined with methotrexate or another disease-modifying drug. There is evidence that both of these approaches are effective in reducing synovitis, limiting erosive damage, and improving quality of life.

Combining conventional disease-modifying drugs has one major drawback: it increases toxicity. Many of the earlier combinations, particularly those involving injectable gold, had too much toxicity to make them useful in clinical practice. Triple therapy using methotrexate, sulfasalazine, and hydroxychloroquine is considered to have the best efficacy and the least toxicity ^12^. It has become the most widely used conventional combination. The efficacy of disease-modifying drug combinations can be enhanced by short-term treatment with steroids, including intramuscular injections of methylprednisolone.

Early biologics, particularly TNF inhibitors such as infliximab, were given in combination with methotrexate because this approach improved sustained efficacy. Most biologics are now combined with methotrexate. There is evidence that some biologics, particularly the interleukin-6 inhibitor tocilizumab, might also be effective in monotherapy when methotrexate is contraindicated. There is limited evidence that other disease-modifying drugs are beneficial when used with biologics. However, when TNF inhibitors are combined with triple therapy (methotrexate, sulfasalazine, and hydroxychloroquine), more patients remain on their biological treatment ^13^.

Strategy trials show that intensive combinations using combinations of conventional disease-modifying drugs have broadly similar efficacy to combination using biologic agents, particularly TNF inhibitors with methotrexate. The relative benefits of combining methotrexate with other disease-modifying drugs or with high-dose, short-term glucocorticoids in early disease are contentious, and different trials provide varying perspectives ^14, 15^. The main benefit of combinations of conventional disease-modifying drugs is that they provide similar improvements in quality of life but cost substantially less. As a consequence, they would invariably be preferred when viewed from a cost-effective perspective. However, biologic combinations are more rapidly effective and have less toxicity. The introduction of biosimilar biologics may change the balance of health economic benefit. Some recent trials comparing different strategies are summarised in Table 1 ^16– 20^.

**Table 1.** Trials comparing intensive treatment strategies using conventional and biologic drugs.

Trial	Year	Type	Patients	Endpoint	Combination Therapy		Primary Outcome	Comparison of Groups
					*Conventional*	*Biologic*
BeST ^16^	2007	Early RA	508	2 years	DMARDs/tapered high-dose prednisone	DMARDs/infliximab	Change in HAQ and X-ray score	Similar
Tear ^17^	2012	Early RA	755	2 years	Triple therapy	Methotrexate/etanercept	DAS28 from 12–24 months	Similar
Swefot ^18^	2012	Methotrexate Failure	258	2 years	Triple therapy	Methotrexate/infliximab	Change DAS28	Similar
RACAT ^19^	2013	Methotrexate Failure	353	1 year	Triple therapy	Methotrexate/etanercept	Change DAS28	Similar
TACIT ^20^	2015	Established RA	205	1 year	Any DMARDs	DMARD/TNF inhibitor	Change HAQ	Non-inferior

BeST and Tear trials had four groups, but only comparison of combination DMARDs and DMARD/biologics has been reported in this table. Triple therapy: methotrexate, sulfasalazine, and hydroxychloroquine. Abbreviations: DAS28, disease activity score for 28 joints; DMARDs, disease-modifying anti-rheumatic drugs; HAQ, health assessment questionnaire; RA, rheumatoid arthritis; TNF, tumour necrosis factor.

The various strategy trials have so far failed to resolve a number of critical uncertainties about treatment intensity. In established RA, when patients have failed to respond to at least one conventional disease-modifying drug, such as methotrexate, there is some doubt about whether it is best to try a combination of conventional disease-modifying drugs or to start biological treatments. From the perspective of healthcare funders, there is much to be said about starting combinations of conventional drugs followed by biologics in patients who fail to respond. In these patients, the slower onset of action of conventional drugs is of limited consequence. As the use of combinations of conventional drugs increases, this issue becomes less crucial, as most patients with severe disease will have already received this lower cost form of intensive therapy.

The main area in which there is debate concerns the initial treatment of RA. Many experts believe that patients with early RA need to be treated with methotrexate monotherapy before receiving other treatments. This perspective is part of existing guidance in North America and much of Europe ^21, 22^. Other guidance, notably from the National Institute for Health and Clinical Excellence in England, recommends starting combinations of conventional disease-modifying drugs in all patients with early disease when first seen by specialists ^23^. In addition, there is considerable evidence from clinical trials that patients with early RA benefit substantially from early intensive treatment with biologics ^24^.

The rationale for starting patients on methotrexate monotherapy is that many patients respond well to this treatment and that using it initially reduces the risks of adverse events and is less expensive. The argument in favour of starting with initial intensive management strategies is that there is an extensive evidence base from clinical trials that this approach is more effective and gives less long-term erosive progression. Given the perspective that patients with early RA need rapid intervention with effective treatments, it seems illogical to start with combinations of conventional disease-modifying drugs when biologics act so much more rapidly. Nevertheless, the expense of biologics probably means they will not be universally used for some time yet. In the fullness of time, it seems inevitable that their use as a first treatment in early RA will gradually increase, though both health economic factors and concerns about risks such as more infections will limit the extent of any changes.

### Bespoke management

RA is a variable disease. In some patients it is mild, and in others it is severe. Not all patients need identical treatment. It therefore seems self-evident that we should move on from standard care, aimed at all patients, towards individualised care: in other words, from “one size fits all” into the realm of bespoke care ^25^. There are several examples of known factors that predict the need for more intensive management. Firstly, almost all drug trials enrol patients with active RA who have high DAS28 scores and many tender and swollen joints. The exact dividing line between active and inactive RA is not well defined. However, it usually involves patients having at least three swollen and tender joints and some evidence of an elevated ESR or C-reactive protein level. Patients with inactive disease do not usually have their treatment changed unless they have adverse events with one drug and need an alternative agent.

**Figure 1.**
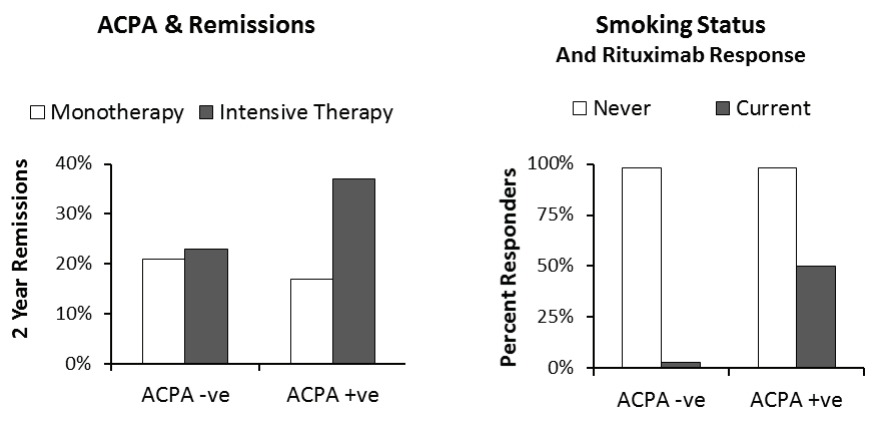
Potential markers of individualised responses. ( **a**) Impact of anti-citrullinated protein antibody (ACPA) status on remissions in early rheumatoid arthritis trial ^26^ showing benefit of intensive treatment restricted to ACPA-positive patients. ( **b**) Impact of smoking status with ACPA positivity during rituximab therapy showing only non-smokers had major benefit ^30^.

There is some evidence that patients who are seropositive for anti-citrullinated protein antibody (ACPA) are more likely to benefit from intensive treatment. Secondary analysis of early RA trial data has shown that intensive treatment is only beneficial in ACPA-positive patients ^26^. The impact of ACPA status on remissions with intensive treatment is shown in Figure 1. It is likely that other markers of severe disease also help identify those patients most likely to benefit from higher treatment intensities. However, there is also evidence that low-risk early arthritis patients benefit from bridging therapy with glucocorticoids given together with methotrexate ^27^. This more recent research implies current assessments of prognostic risk are incomplete, and for the present every patient with early RA could benefit from some form of initial intensive treatment.

There is more evidence about prognostic biomarkers for erosive progression in RA. Patients at higher risk of subsequent erosive disease can be prospectively identified using matrices of different biomarkers and clinical measures ^28, 29^. It is likely that the identification of these higher risk patients using immunological and other markers will help guide treatment decisions in the years ahead.

Some lifestyle factors have also been implicated. Smoking status is the best known of these factors. Observational study data have suggested that smokers are less likely to respond to treatments such as biologics ^30^, which is also shown in Figure 1. However, the information about the negative effects of smoking is not fully evaluated. So far, there have been no suggestions that smokers should be treated differently from non-smokers.

It seems inevitable that in the fullness of time a range of predictive factors for drug treatment will be known. At present, there is insufficient information to know how much benefit this will bring to disease management. It is without doubt an area of intense research interest but so far it remains outside routine practice.

### Impact on new treatments

We are moving beyond the present biologics era with the development of new orally acting drugs. Kinase inhibitors are likely to become an important new class of drugs. Although they have been evaluated using the historic approach of assessing with the conventional core data set measures, there are moves to provide information about their ability to induce remissions. Extending the evidence base in this way is an encouraging innovation.

## Future developments

Despite the impossibility of predicting the future, it remains tempting to suggest how research will develop. At present, it seems inevitable that the current focus on early intensive treatment for RA will continue and, if anything, increase.

**Figure 2.**
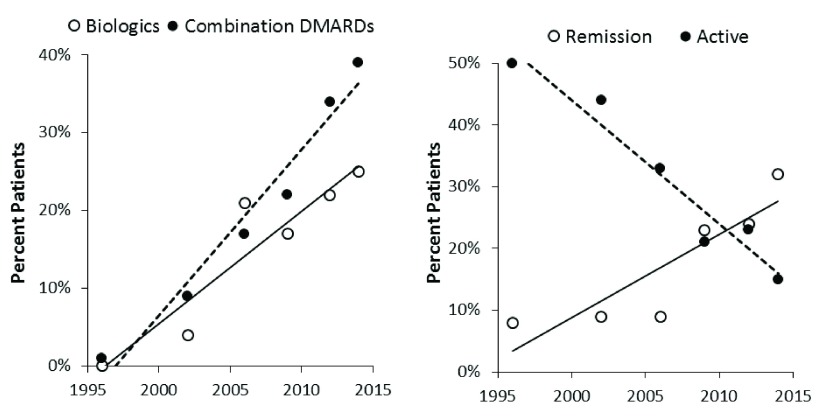
Temporal changes in treatment intensity, remission, and active disease from South London specialist centres showing increase in remissions as treatment intensity increases in routine practice ^31^. Abbreviations: DMARD, disease-modifying anti-rheumatic drug.

The main focus of this development is likely to be further efforts to induce remission in early disease by optimising the initial treatments used. One way to achieve this goal is to start with high-intensity treatment and follow this with lower-intensity maintenance therapy. I believe this is a likely development from the current use of combinations of conventional and biological drugs. There is evidence that this approach is increasing remissions and reducing active disease in routine practice. Evidence supporting this view from my own unit is shown in Figure 2 ^31^.

Another focus is identifying patients with RA as soon as possible and even screening for “pre-rheumatoid” patients who are positive for ACPA but currently do not have definite RA. There is some evidence that treating these patients with disease-modifying drugs or steroids can prevent the onset of RA. There are currently major difficulties in identifying and classifying them. However, this focus on earlier and earlier disease is changing perspectives. There is also some evidence, albeit incomplete, that they are also improving outcomes.

Over the four decades I have been involved in treating RA, there have been major changes in its management and substantially better outcomes have been achieved. The pace of change continues and, as a consequence, to my mind, RA appears to be a far less onerous burden for patients. However, from the perspective of individual patients, this benefit is less obvious because the impact of a moderate disease upon everyday lives is not much less than the impact of a severe disease. This apparent paradox means that there is still a long way to go in improving the lives of people with arthritis.
